# The Future Life

**Published:** 1893-04

**Authors:** 


### THE FUTURE LIFE.

In an article by Archbishop Farron on "Conceptions of a Future Life," in the North American Review, for March, he says : The separate existence of the soul has been as much the absolute conviction of the supremest intellects, which have shown upon the world, as of the humblest and most illiterate peasants. The Cogito ergo sum of

Descartes is unanswerable. To attribute the illimitable range and diversities of thought is nothing more than infinitesimal molecular changes in the grey substance of the brain is the most miserable, absurd, unverifiable, and imposssible of all guesses. Mr. Bain may well acknowledge the difficulty of "storing up in three pounds' weight of albuminous and fatty tissue all of our acquired knowledge!" All mankind then, except perhaps one in every ten millions, will admit that we have souls, and that essentially we are souls. But what is the soul? This question has agitated all philosophy, heathen as well as Christian. Heathen philosophy had nothing but the merest empiricism to offer in its solution. "Quid sit porro ipse animus, aut ubi, aut unde, magna dissensio est," says Cicero in his Tusculan questions. No wonder, therefore, that some philosophers believed that the soul perished with the body; others that it lasted for a time and then was dissipated; others that it continued forever. As to its localization, Aristotle placed it in the heart; Empedocles in the pericardium; others, like our modern materialist, identified it with the brain; to others again the soul (animus) was but the breath (anima). Zeno thought that it was a breathing fire. Aristoxenus vaguely declared that it was a harmony (ipsius corporis intentio); Democritus, that it resulted from a fortuitous concourse of atoms; Xenocrates, following Pythagoras, defined it, not very luminously, as "a self moving member." Plato analyzed it into the Reason, the Passion and the Desires. Aristotle thought that it was a sort of fifth essence, to which he gave the name Eutelechy—a name which so puzzled Hermolaus Barbarus that he is said to have evoked thc Demon to tell him its true significance!

